# Potential Risk Factors Associated with Infection with Bovine Leukaemia Virus in Dairy and Beef Cattle in Taiwan

**DOI:** 10.3390/pathogens10121553

**Published:** 2021-11-29

**Authors:** Yi-Chen Chen, Wen-Yu Chin, Chao-Chin Chang, Shih-Te Chuang, Wei-Li Hsu

**Affiliations:** 1 Division of Endocrinology and Metabolism, Department of Internal Medicine, Taichung Veterans General Hospital, Taichung 407, Taiwan; tyler7418@hotmail.com; 2 Department of Farm Animal Health, Faculty of Veterinary Medicine, Utrecht University, 3584 CS Utrecht, The Netherlands; kellywychin@gmail.com; 3 Graduate Institute of Microbiology and Public Health, College of Veterinary Medicine, National Chung Hsing University, Taichung 402, Taiwan; changcc@dragon.nchu.edu.tw; 4 Department of Veterinary Medicine, College of Veterinary Medicine, National Chung Hsing University, Taichung 402, Taiwan; stchuang@dragon.nchu.edu.tw

**Keywords:** bovine leukaemia virus, dairy cows, beef cattle, heifer, risk factors

## Abstract

Bovine leukaemia virus (BLV), which is classified as a *Deltaretrovirus*, is the aetiologic agent of enzootic bovine leukosis (EBL), a chronic lymphoproliferative disorder with a worldwide distribution. EBL is widespread in dairy herds and causes a direct economic impact due to reduced milk production and the early culling of BLV-infected cattle. The BLV infection status in Taiwan remains largely unknown; a high prevalence of BLV in dairy cows was recently revealed. The present study further investigated BLV infections in beef cattle. Surprisingly, the prevalence of BLV proviral DNA was as low as 11.8% (23/195), which is significantly lower than that noted in dairy cows, which was 42.5% (102/240) (*p* < 0.001). Factors associated with BLV infections were subsequently investigated. Due to the differences in herd management, an analysis of risk factors for a BLV infection was independently conducted in these two sectors. Several factors associated with a BLV infection were identified. Age was significantly associated with BLV infection status in dairy cows (*p* < 0.001) but not in beef cattle. A high prevalence of BLV was observed in cattle >15.5 months old (57.8%) compared with those ≤15.5 months old (11.4%). Moreover, after stratification analysis, based on the critical age of 15.5 months, as determined by the receiver operating characteristic (ROC) curve, a significantly higher BLV prevalence was demonstrated in lactating dairy cows, cattle undergoing bull breeding, heifers at older ages, and those undergoing routine rectal palpation. Due to the high prevalence of BLV in Taiwan, the development of an effective control program, based on the identified risk factors, is important for interrupting the routes of BLV transmission within herds.

## 1. Introduction

Bovine leukaemia virus (BLV), belonging to the *Deltaretrovirus* genus of the *Retroviridae* family, is the causative agent of enzootic bovine leukosis and is the most common neoplastic disease of cattle. BLV infection is mostly asymptomatic, but in less than 5% of infected cattle, it manifests as a malignant B-cell lymphoma [1]. Additionally, a negative association has been reported between herd-level milk production and herd BLV prevalence in Canadian dairy herds [2,3]. Moreover, BLV infection has a negative impact on cow longevity [4,5] and restricts the international trade of infected animals. In addition to affecting cattle, reports from Buehring and colleagues indicate the potential public health implications of BLV in humans, as evidenced by the presence of the BLV antibody (capsid antigen, p24) in 74% of the sera [6], and proviral DNA in 44% of breast tissue [7]. However, whether BLV may play a direct role in human diseases requires further investigation.

BLV is widespread in domestic cattle worldwide, and a high prevalence of BLV infection has been reported in many countries, including the US, Canada, Japan, and China, that lack BLV-specific eradication programs [8,9,10,11]. It is reported that horizontal transmission, including direct contact and indirect (iatrogenic) contact with blood or, less likely, with milk from BLV-affected animals, is the common mode for spreading BLV to susceptible individuals in cattle populations [12]. In particular, BLV is possibly spread by iatrogenic transmission via the use of blood-contaminated needles, instruments, or gloves in medical management, dehorning, or rectal palpation, respectively. Moreover, vertical transmission, such as the transplacental route, of BLV could also be possible [13,14]. Nevertheless, the contribution of the aforementioned modes of transmission could depend on the frequency and nature of BLV exposure and needs to consider the prevalence of BLV within the herds [15]. Given that BLV infection is life-long, and that persistent infection leads to a continuous detrimental effect [16,17], it is necessary to determine the modes of transmission for the control of BLV infection so as to properly intervene.

Very recently, a nationwide surveillance of BLV in dairy cows was conducted in Taiwan, and the seroprevalence of BLV at the animal and herd levels was as high as 81.8% and 99.1%, respectively [18]. However, the infection status in beef remains unknown. Notably, a national control program for BLV infection in dairy cows has not yet been implemented in Taiwan, potentially because of the lack of risk factor analyses for BLV infection. Hence, the main objective of this study was to identify the potential risk factors associated with cow-level BLV infection in cattle in Taiwan. In this study, the infection status in beef cattle was investigated for the first time, and the risk factors for beef and dairy cows were comparatively analysed.

## 2. Results

### 2.1. Prevalence and Genotype of BLV Env Proviral DNA in Beef and Dairy Cows

Given that BLV prevalence in beef cattle in Taiwan remains unknown, the BLV infection status in beef cattle was initially investigated by nested PCR. Of the 195 blood samples, the prevalence of BLV proviral DNA in beef was 11.8% (23/195). Moreover, a sequence analysis of the partial *env* gene, amplified from beef cattle, indicated that all samples analysed were BLV genotype 1.

**Figure 1:**
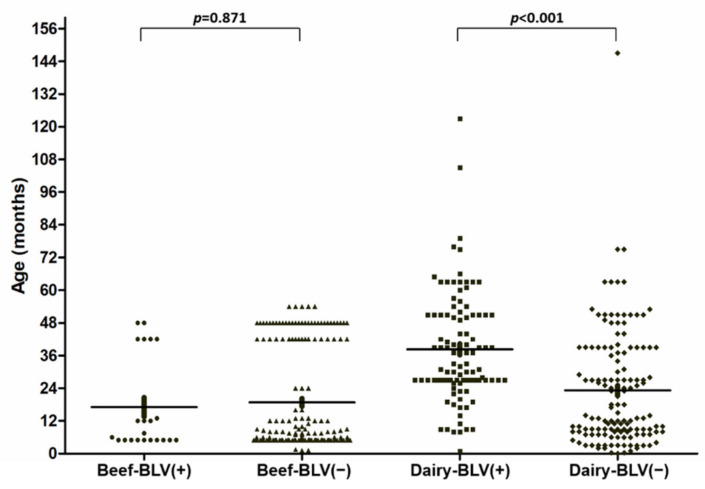
Distribution of ages in beef (● and ▲) and dairy cows (■ and ◆). The age composition in the group of beef or dairy cows infected with BLV (+) or without BLV (−) were plotted.

Notably, the BLV prevalence in beef cattle (11.8%) was significantly lower than that in dairy cows (42.5%; 102/240) (*p* < 0.001). It has been demonstrated that the incidence of BLV in beef and dairy cows increases with age [19]. However, no significant difference in the mean age of beef cattle infected with BLV compared with beef cattle without BLV infections was noted (Figure 1, 17.07 ± 3.49 vs. 19.08 ± 1.41 months old, respectively, *p* = 0.871). Nonetheless, the mean age of dairy cows with a BLV infection was significantly older than that of BLV-negative dairy cows (Figure 1, 38.28 ± 1.99 vs. 23.20 ± 1.80 months old, respectively, *p* < 0.001).

### 2.2. Distributions of BLV Proviral DNA Prevalence among the Farm Features of Beef and Dairy Cows

The prevalence of BLV infection in all cows enrolled in this study was further analysed based on the characteristics of farm animals. As listed in Table 1, in addition to older age, positive BLV detection occurred more often in dairy cows (*p* < 0.001), Holstein breeds (*p* = 0.001), female cattle (*p* < 0.001), herds over 400 head (*p* = 0.015), intensive management (free-stall housing) (*p* = 0.004), farm 6 (*p* = 0.015), cattle breeding via artificial insemination (*p* < 0.001), cattle with rectal palpation (*p* < 0.001), and cattle that were fed sterilized colostrum (*p* = 0.008).

Considering the differences in herd management, the cattle were stratified into beef and dairy cows to investigate the potential risk factors associated with BLV infection (Table 2). Interestingly, none of the farm factors significantly correlated with BLV status in beef cattle. However, in dairy cows, BLV prevalence was significantly associated with farm size (*p* = 0.003), breeding technique (*p* < 0.001), and cattle undergoing routine rectal palpations (*p* < 0.001).

**Figure 2:**
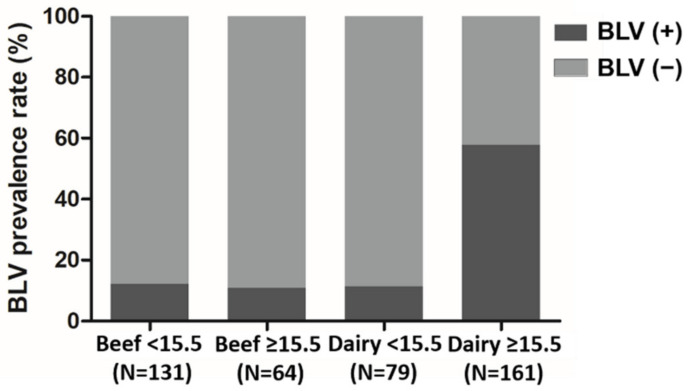
Prevalence of BLV in beef and dairy cows, divided by age. The cut-off value of age (15.5 months) was determined by ROC curve analysis. The status of BLV proviral DNA of each age group in beef and dairy cows is shown individually.

To evaluate the potential confounding effect due to age, the dairy cows were stratified into two groups using the age of 15.5 months as a cut-off, based on ROC curve analysis (Figure 2). As summarized in Table 3, lactating cows accounted for 96.3% (155/161) of dairy cows older than 15.5 months, which is the optimum age for breeding in reproductive management, and almost all the dairy cows that were ≤15.5 months were heifers (98.7%, 78/79). Notably, BLV prevalence increased with age in cattle; BLV proviral DNA was detected in 57.8% (93/161) and 11.4% (9/79) of cattle in the >15.5 months and ≤ 15.5 months groups, respectively (*p* < 0.001). In contrast, the BLV prevalence was generally equal for these two age groups of beef cattle (i.e., 12.2%, 16/131; 10.9%, 7/64, respectively, *p* = 0.795). Moreover, in dairy cows older than 15.5 months, BLV infection was significantly associated with the factors of having been bred with both artificial insemination and bulls (*p* < 0.001), receiving rectal palpation (*p* < 0.001), and not having a vaccination history (*p* < 0.001).

### 2.3. Differences of BLV Expression among the Farm Features of Lactating Dairy Cows and Heifers

BLV activation may be induced when dairy cows encounter internal stressor stimuli, such as the lactation cycle [20], and herd management of lactating cows is distinct from that of heifers and might be involved in the variable susceptibility to BLV infection. Thus, the dairy cows were further classified into groups of lactating dairy cows and heifers (Table 4). Results indicated that the BLV infection rate was significantly higher in lactating dairy cows that were either bred with bulls (*p* = 0.004) or were from farm 3 (*p* = 0.038) (Table 4). Notably, age was the only farm factor correlated with BLV infection in heifers. As very limited factors were involved in the BLV infection, multivariate analysis was not further conducted.

### 2.4. Prevalence of BLV Env Proviral DNA in the Breeding Bulls, Lactating Dairy Cows and Heifers from Farm 3

It is worth noting that the BLV prevalence in lactating cows originating from farm 3 was significantly higher (80%, 24/30) than that in other farms (58.6%, 46.7%, and 52.2% for farms 4, 5, and 6, respectively, *p* = 0.038, C.I. = 0.031–0.040) (Table 4). To investigate the possibility of vertical transmission of BLV in the farm, we further collected blood samples from all breeding bulls (12 cattle) and four offspring delivered by the dairy cows that were analysed in this study. BLV proviral DNA was detected in 41.7% (5/12) of breeding bulls, whereas none of the offspring were infected by BLV.

Moreover, to investigate the prevalence of BLV in milk, both milk and blood samples were randomly collected from 31 lactating dairy cows on farm 3, and BLV proviral DNA was simultaneously detected. The prevalence of BLV env proviral DNA was 90.3% (28/31) in blood samples and 29.0% (9/31) in milk samples, respectively. It was further found that the testing results of BLV proviral DNA positivity between milk samples and blood samples were not statistically consistent (McNemar’s test, *p* < 0.05).

## 3. Discussion

Due to the high prevalence of BLV in dairy cows in Taiwan, it is urgent to identify the primary route of BLV transmission within herds, which could facilitate the formulation of management strategies to control the spread of BLV. Although risk factors for BLV infection have been well documented, the studies were from various regions and continents worldwide. Considering that herd management and medical practices can vary among countries, this study determined the prevalence of BLV proviral DNA in beef cattle and evaluated the risk factors associated with BLV infection between beef and dairy cows in Taiwan for the first time. The prevalence of BLV infection in beef cattle was 11.8% (23/195), which is significantly lower than that of dairy cows (42.5%, *p* < 0.001). Although all dairy cows were female, the sex of the cattle was not associated with BLV prevalence in beef. Specifically, in the case of female beef (n = 68), the BLV prevalence was as low as 10.2% (7/68), which is similar to the overall prevalence (11.8%) in the beef group. Nevertheless, our results strongly indicated that dairy cows are significantly more susceptible to BLV infection than female beef (Supplementary Table S1). Noticeably, the BLV infection rate in dairy cows varied among the herds analysed in this study. Dairy cows with older ages had a higher BLV prevalence, and a higher BLV prevalence in lactating dairy cows was positively associated to cattle receiving bull breeding (*p* < 0.001), heifers at older ages (*p* < 0.001), and routine rectal palpations (*p* < 0.001) (Table 3). In contrast, none of the parameters analysed herein were associated with BLV prevalence in beef cattle.

The prevalence of BLV proviral DNA in the present study was considerably lower in beef cattle than in dairy cows, which was consistent with other reports originating from other countries, such as Japan [21] and the United States [5]. Certain differences were indicated between these two sectors. First, the overall health plan (e.g., vaccination programs) and herd management practices (e.g., frequency of pregnancy examination) differed for dairy versus beef cattle. In addition, the immune response and susceptibility to pathogens of dairy cows might differ from those of beef cattle and may be attributed to inherent variability [22]. According to these intrinsic differences and distinct herd management practices between dairy and beef cattle, and even between herds with the same production purpose, the two sectors might not be exposed to the same risk factors at an equivalent frequency. Noticeably, although no parameters associated with BLV prevalence were identified in beef cattle, based on the stratification analysis (Table 2), the presence of BLV proviral DNA in dairy cows was positively associated with age, herd size, breeding method, and rectal palpation (Table 2).

It has been demonstrated that the prevalence of BLV gradually increased with age in dairy cows, and the odds ratio for BLV infection in dairy cows ≥6 years old was increased 2.3-fold, compared with that noted for dairy cows ≤1 year old [19]. Similarly, Gutiérrez et al. monitored BLV progression from birth until the first lactation in a farm where management procedures for the prevention of blood contact were fully implemented. The results indicated that BLV infection was undetectable in cattle from birth to 12 months, whereas proviral DNA was detected from 15 months of age and increased with age [15]. In our study, as indicated in Figure 2, the prevalence of BLV in cattle under the age of 15.5 months did not differ between beef (11.8%, 15/127) and dairy cows (11.3%, 8/71), whereas the overall BLV prevalence in dairy cows (42.5%) was much higher than that in beef cattle (11.8%). Notably, the mean age of beef cattle (18.62 ± 18.41 vs. 29.61 ± 21.94, *p* < 0.001) was much younger than that of dairy cows; hence, it is possible that the age of animals enrolled in this study biased the BLV prevalence in these two sectors. Indeed, based on the cut-off value (i.e., 15.5 months) determined by the ROC analysis, BLV prevalence in two age groups was further assessed. The prevalence of BLV proviral DNA was 11.4% and 57.8% in the dairy cows at ages younger and older than 15.5 months, respectively (Figure 2), indicating that the risk of BLV infection increased significantly with age in dairy cows (*p* < 0.001). The cows were accommodated in free-stall housing, and given that horizontal transmission via direct contact or indirect exposure (e.g., iatrogenic procedures) of susceptible animals to the infected blood is reported as the major route of BLV spread [12], one can expect the chance of BLV contact and the level of BLV viral load in cattle to increase over time, thus ultimately increasing the risk of infection. Moreover, several lines of evidence indicated blood-sucking insects, such as stable flies and horn flies, as a risk factor of BLV transmission under experimental conditions [23,24]. Hence, an increase of the frequency of exposure to mechanical transmission vectors could also contribute to the higher BLV prevalence in older cows.

Based on body size, most dairy heifers reach puberty when cattle undergo regular breeding procedures at the age of approximately 15 months [15]. Thereafter, the dairy cows were further divided into heifer and lactating dairy cows to adjust for the possible impact of age on BLV infection. As indicated in Table 4, age was the only risk factor for BLV infection in dairy heifers following subgrouping. Notably, BLV positivity was detected only in 11.5% of heifers (9/78) <15.5 months old, which was significantly lower than that noted in those >15.5 months old (50%, 3/6). Noticeably, all heifers older than 15.5 months (N = 6) originated from farm 3, and three out of the six heifers with BLV positivity were bred using artificial insemination and rectal palpation. Single-use rectal gloves during pregnancy examination could possibly avoid virus spreading carried over from the infected cows previously examined, given that the number of palpations conducted with the same gloves was positively correlated with BLV positivity [12]. Moreover, older heifers (ranging 17-21 months old) and breeding with bulls might reflect that breeding management was not well controlled on farm 3. These factors could all contribute to the remarkably higher BLV prevalence in dairy cows on farm 3.

In addition, an apparently higher BLV prevalence was noticed in lactating dairy cows that were bred with bulls (88%, 22/25). BLV transmission occurs mainly through the transfer of BLV-containing lymphocytes present primarily in blood, colostrum, and milk [25], and potentially in other biological fluids, such as the semen of BLV-infected bulls [26]. The role of breeding bulls in BLV transmission has been reported [27]. Natural services may facilitate BLV dissemination via trauma during copulation, resulting in the transfer of blood between infected and uninfected animals. As evidenced by the presence of BLV proviral DNA, in addition to blood, semen, or smegma, could also mediate BLV dissemination to recipient cows [27]. However, very recently, a report from Benitez et al. demonstrated that the natural breeding of cattle does not represent a route for BLV transmission, based on studies monitoring the BLV infection status of heifers co-housed with BLV-affected bulls [28]. Given that the study was performed over a 38-day co-housing period during a defined breeding season, the contact frequency and the BLV proviral load of bulls should be also taken into account when evaluating the possible contribution of natural breeding with bulls to BLV transmission in cows. In the current study, bulls from farm 3 were purchased from another farm, and BLV proviral DNA was detected in five out of twelve bulls (41.7%). Considering that the exposure of susceptible animals to infected blood is one of the major methods for the spread of BLV [12], confirmation of the BLV-free status of new cattle (e.g., bulls or calves) purchased from other farms, and of herd bulls prior to the breeding season, could be beneficial for mitigating the spread of the virus within the herd.

In this study, BLV prevalence in beef cattle was depicted, and for risk assessment, BLV proviral DNA in dairy cows was simultaneously detected. However, as described herein, BLV prevalence in dairy cows is relatively lower than that reported in one previous study [18]. Notably, the experiment design differed in these two studies. In the current study, to determine the relation of age with BLV prevalence, we purposely collected cattle at different age groups, including 84 young heifers, for which BLV prevalence (11.4%) was significantly lower than cows in the older age group. However, in the previous study, six cattle were randomly selected from one herd. Since the previous study was unable to track the ages of cattle, we cannot measure the contribution of the sampling difference to the variation of BLV prevalence in the two studies. Moreover, the high prevalence of BLV in Taiwan has been noted since the first nationwide BLV surveillance, conducted in 2016 [18]. Hence, we suspected, in addition to the aforementioned sampling bias, that the lower BLV prevalence could be possibly due to the effectiveness of prevention propaganda, such as the use of single-use rectal gloves for palpation, using one needle per cow for vaccination, etc.

## 4. Materials and Methods

### 4.1. Sample Collection

A cross-sectional study was carried out on cattle in four major counties with beef farming in Taiwan in 2019. The minimal sampling number of cattle and herd for prevalence estimation were determined based on the assumptions of 95% confidence, 5% error rate of the prevalence, and BLV seroprevalence of 81.8% and 99.1% at the animal and herd levels, respectively, according to the previous study in Taiwan [18].

**Figure 3:**
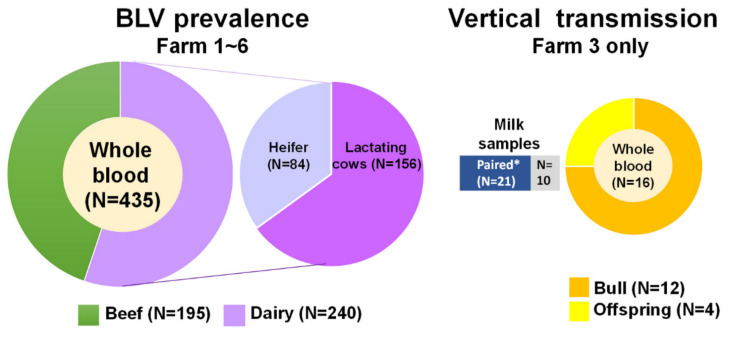
Samples enrolled in this study. For analysis of BLV prevalence, a total of 435 whole blood samples were collected from 195 beef cattle and 240 dairy cows, including lactating dairy cows (n = 156) and heifers (n = 84), from six herds in central and southern Taiwan. Moreover, for the vertical transmission study, 16 whole blood samples were obtained from 12 breeding bulls and 4 offspring, as well as 31 milk samples collected from 31 lactating dairy cows from farm 3, located in southern Taiwan. Notably, 21 out of 31 milk samples were collected from the lactating cows enrolled in the horizontal study (i.e., pair sample, indicated as paired *).

There are two sets of experiments: (I) initially, for the evaluation of BLV proviral DNA prevalence, a total of 435 whole blood samples were drawn in EDTA tubes from 195 beef cattle and 240 dairy cows composed of lactating dairy cows (n = 156) and heifers (n = 84) of six herds in central and southern Taiwan (Figure 3, and Table 5). (II) For the evaluation of the possibility of vertical transmission, as well as the detection of the BLV in milk, 16 whole blood samples were obtained from 12 breeding bulls and 4 offspring delivered by the cows analysed in this study, as well as 31 milk samples collected from 31 lactating dairy cows from farm 3, located in southern Taiwan (Figure 3).

All samples were obtained from healthy cattle without clinical signs of disease that were evaluated by experienced veterinarians, based on the criteria used in a previous study [18], and with the owner’s consent. The medical records of each cattle were collected during the farm visit for further statistical analysis. To this end, a questionnaire was designed to collect data on cattle information, including the species, age, breed, and gender of the cattle, and the herd size of the farm, as well as the herd health-management practices, including the methods of artificial insemination, frequency of rectal palpation, colostrum condition, and records of vaccination, etc., as listed in Table 1.

### 4.2. DNA Extraction and Detection of BLV Proviral DNA by Nested PCR

Genomic DNA was extracted from the leukocytes fractionated from 5 mL of anticoagulated blood samples, and from milk somatic cells isolated from 15 mL of raw milk, by using the AllPure Genomic DNA Kit (Universal, AllBio, Taichung, Taiwan) according to the manufacturer’s instructions. DNA samples were stored at −20 °C until polymerase chain reaction (PCR) was conducted. The quantity of DNA was measured by spectrophotometry (NanoDrop Lite, Thermo Scientific, Waltham, MA, USA), and the integrity of DNA was further validated by PCR with a set of primers for the amplification of bovine glyceraldehyde 3-phosphate dehydrogenase (*GAPDH*), which was used in the previous report [29].

The BLV proviral DNA was detected by nested PCR via the amplification of the partial env gene with two sets of primers, designated as BLV-env-1 5′-TCT GTG CCA AGT CTC CCA GAT A-3′ (outer forward) and BLV-env-2 5′-AAC AAC AAC CTC TGG GAA GGG-3′ (outer reverse), and BLV-env-3 5′-CCC ACA AGG GCG GCG CCG GTT T-3′ (inner forward) and BLV-env-4 5′-GCG AGG CCG GGT CCA GAG CTG G-3′ (inner reverse), which were described previously [18]. The PCR conditions of BLV env amplification were set according to a previous study [30]. For the genotyping of BLV, the identity of the env amplicons was confirmed by an automated Sanger’s sequencing method (Mission Biotec, Taipei, Taiwan).

### 4.3. Statistical Analysis

The differences in BLV proviral DNA positivity detected from the whole blood samples of various types of cattle (beef cattle, lactating dairy cows, or heifers), and the different features of cattle, such as species, breed, gender, the farm from where those cattle originated, and herd size, as well as management procedures, including artificial insemination, rectal palpation, sterilized colostrum feeding, and vaccination conditions, were comparatively analysed via the Chi-square test. However, owing to the limited study size, the difference in BLV proviral DNA positivity by different farm management patterns was evaluated by Fisher’s exact test. In addition, to compare the mean age difference between BLV proviral DNA positive and negative animals, the Mann–Whitney U test was applied because normal distributions were not indicated among these two groups. Those explanatory variables with significant difference (*p* < 0.05) were then selected for the multivariate analysis. Risk evaluation for the relationship between age and the presence of BLV proviral DNA was determined by receiver operating characteristic (ROC) curve analysis. The critical age for further stratification analysis was determined by the maximized sum of sensitivity and specificity from the ROC curve. The consistency of BLV proviral DNA positivity between blood samples and milk samples was evaluated by McNemar’s test. The statistical analysis was conducted with the Statistical Package for the Social Sciences (SPSS Version 22, Asia Analytics Taiwan, Ltd. Taipei, Taiwan.) All *p* values were carried out with two-tailed tests, and the statistical significance was determined by *p* values less than 0.05.

## 5. Conclusions

To date, eradication programs of BLV have not yet been established nationwide in Taiwan. This study reported, for the first time, the prevalence of BLV infections in beef cattle in Taiwan, and identified several within-herd factors, including age, cattle breeding using bulls, and routine rectal palpation, which are positively associated with BLV infections. BLV is widespread in Taiwan. The limitation of this study was due to the unknown BLV prevalence, and the sampling design for beef cattle was based on dairy cows, which led to a small sample size of beef cattle. Despite the small scale of this investigation, the findings may inform the formulation of preventive measures for reducing the clinical impacts of BLV infection in certain contaminated farms, particularly when a nationwide risk assessment is not available.

## Figures and Tables

**Table 1:** Univariate analysis of the association of farm features with BLV infection of all cattle.

Factors	BLV Detection		
	Positive (*n* = 125)	Negative (*n* = 310)	*p*-Value
**Purpose of production**			<0.001 ^§^
Beef	23	172	
Dairy	102	138	
**Age (months)**	34.38 ± 1.90 ^†^	20.78 ± 1.13 ^†^	<0.001 ^‡^
**Breed**			0.001 ^§^
Holstein	118	249	
Angus	6	55	
Mixed	1	6	
**Gender**			<0.001 ^§^
Male	13	97	
Female	109	199	
Castrated male	3	14	
**Herd size (head)**			0.015 ^§^
<50	19	47	
50–200	5	45	
201–400	97	205	
>400	4	13	
**Management pattern**			0.004 ^¶^
Intensive	123	281	
Pasture	2	29	
**Farm owner**			0.015 ^§^
1	4	13	
2	2	29	
3	34	70	
4	27	89	
5	18	42	
6	40	67	
**Artificial insemination**			<0.001 ^§^
No	20	139	
No (Bull only)	28	58	
Yes	63	100	
Yes (Combined)	14	13	
**Regular rectal palpation**			<0.001 ^§^
No	29	196	
Yes	96	114	
**Sterilized colostrum**			0.008 ^§^
No	6	42	
Yes	119	268	
**Vaccination**			0.865 ^§^
No	17	43	
Yes (Needle sharing)	66	171	
Yes (One shot per needle)	42	96	

^†^ The values represent means ± SEM. Differences of analytes within groups and BLV positivity was performed with the ^‡^ Mann–Whitney U test, ^¶^ Fisher’s exact test, and ^§^ Chi-square test. BLV, bovine leukaemia virus. *p* < 0.05 indicates significant difference.

**Table 2:** Univariate analysis of the association of farm characteristics with BLV infection of beef and dairy cows.

Factors	BLV in Beef Cattle			BLV in Dairy Cows		
	Positive (*n* = 23)	Negative (*n* = 172)	*p*-Value	Positive (*n* = 102)	Negative (*n* = 138)	*p*-Value
**Age (months)**	17.07 ± 3.49 ^†^	19.08 ± 1.41 ^†^	0.871 ^‡^	38.28 ± 1.99 ^†^	23.20 ± 1.80 ^†^	<0.001 ^‡^
**Breed**			0.841 ^§^			NA
Holstein	16	111		102	138	
Angus	6	55		0	0	
Mixed	1	6		0	0	
**Gender**			0.702 ^§^			NA
Male	13	97		0	0	
Female	7	61		102	138	
Castrated Male	3	14		0	0	
**Herd size**			0.276 ^§^			0.003 ^§^
<50	4	13		0	0	
50–200	2	35		17	12	
201–400	3	27		2	18	
>400	14	97		83	108	
**Management pattern**			0.542 ^¶^			NA
Intensive	21	143		102	138	
Pasture	2	29		0	0	
**Farm owner**			0.514 ^§^			0.216 ^§^
1	4	13		0	0	
2	2	29		0	0	
3	3	27		31	43	
4	10	77		17	12	
5	4	26		14	16	
6	0	0		40	67	
**Artificial insemination**			0.522 ^§^			<0.001 ^§^
No	17	111		3	28	
No (Bull only)	6	55		22	3	
Yes	0	6		63	94	
Yes (Combined)	0	0		14	13	
**Regular rectal palpation**			0.260 ^¶^			<0.001 ^§^
No	21	137		8	59	
Yes	2	35		94	79	
**Sterilized colostrum**			0.862 ^§^			NA
No	6	42		0	0	
Yes	17	130		102	138	
**Vaccination**			0.525 ^§^			0.355 ^§^
No	3	27		14	16	
Yes (Needle sharing)	18	116		48	55	
Yes (One shot per needle)	2	29		40	67	

^†^ The values represented means ± SEM. Differences of analytes within groups and BLV positivity was performed with the ^‡^ Mann–Whitney U test, ^¶^ Fisher’s exact test, and ^§^ Chi-square test. BLV, bovine leukaemia virus. NA, not applicable. *p* < 0.05 indicates significant difference.

**Table 3:** Differences of characteristics between lactating dairy cows and heifers classified by age.

Factors	Age (months)		
	≤15.5 (*n* = 79)	>15.5 (*n* = 161)	*p*-Value
**Type**			<0.001 ^¶^
Lactating	1	155	
Heifer	78	6	
**BLV**			<0.001 ^§^
Negative	70	68	
Positive	9	93	
**Herd size**			<0.001 ^§^
50–200	0	29	
201–400	20	0	
>400	59	132	
**Farm owner**			<0.001 ^§^
3	39	35	
4	0	29	
5	0	30	
6	40	107	
**Artificial insemination**			<0.001 ^§^
No	31	0	
No (Bull only)	0	25	
Yes	47	110	
Yes (Combined)	1	26	
**Regular rectal palpation**			<0.001 ^§^
No	67	0	
Yes	12	161	
**Vaccination**			<0.001 ^§^
No	0	30	
Yes (Needle sharing)	39	64	
Yes (One shot per needle)	40	67	

Differences of analytes within groups and BLV positivity was performed with ^¶^ Fisher’s exact test and a ^§^ Chi-square test. BLV, bovine leukaemia virus. *p* < 0.05 indicates significant difference.

**Table 4:** Univariate analysis of farm characteristics and BLV infection of lactating dairy cows and heifers.

Factors	BLV in Lactating Dairy Cows			BLV in Heifers		
	Positive (*n* = 90)	Negative (*n* = 66)	*p*-Value	Positive (*n* = 12)	Negative (*n* = 72)	*p*-Value
**Age (months)**	41.94 ± 1.94 ^†^	39.98 ± 2.36 ^†^	0.336 ^‡^	10.83 ± 1.41 ^†^	7.81 ± 0.51 ^†^	0.049 ^‡^
**Herd size**			0.911 ^§^			0.722 ^¶^
50–200	17	12		2	18	
>400	73	54		10	54	
**Farm owner**			0.038 ^§^			0.656 ^§^
3	24	6		7	37	
4	17	12		0	0	
5	14	16		0	0	
6	35	32		5	35	
**Artificial insemination**			0.004 ^§^			0.521 ^¶^
No	0	0		3	28	
No (Bull only)	22	3		0	0	
Yes	54	50		9	44	
Combined	14	13		0	0	
**Regular rectal palpation**			NA			0.251 ^¶^
No	0	0		8	59	
Yes	90	66		4	13	
**Vaccination**			0.059 ^§^			0.656 ^§^
No	14	16		0	0	
Yes (Needle sharing)	41	18		7	37	
Yes (One shot per needle)	35	32		5	35	

^†^ The values represented means ± SEM. Differences of analytes within groups and BLV positivity was performed with ^‡^ Mann–Whitney U test, ^¶^ Fisher’s exact test, and ^§^ Chi-square test. BLV, bovine leukaemia virus. NA, not applicable. *p* < 0.05 indicates significant difference.

**Table 5:** Basic information of the beef and dairy cows analysed in this study.

Factors	Farm Owner					
	1 (*n* = 17)	2 (*n* = 31)	3 (*n* = 120)	4 (*n* = 116)	5 (*n* = 60)	6 (*n* = 107)
**Production usage**	Beef	Beef	Mixed	Mixed	Mixed	Dairy
**Types**						
Beef	17	31	30	87	30	0
Dairy	0	0	30	29	30	67
Heifer	0	0	44	0	0	40
Offspring	0	0	4	0	0	0
Breeding bull	0	0	12	0	0	0
**Age (months)**	10.15 ± 0.55 ^†^	48.00 ± 0.00 ^†^	18.87 ± 1.55 ^†^	13.59 ± 1.50 ^†^	46.80 ± 2.63 ^†^	25.89 ± 1.83 ^†^
**Breed**						
Holstein	16	0	120	110	30	107
Angus	0	31	0	0	30	0
Mixed	1	0	0	6	0	0
**Gender**						
Male	0	0	41	81	0	0
Female	1	31	74	35	60	107
Castrated male	16	0	5	0	0	0
**Herd size**	<50	50–200	>400	50–200	>400	>400
**Management pattern**	Intensive	Pasture	Intensive	Intensive	Intensive	Intensive

^†^ The values represented means ± SEM.

## Data Availability

All the data supporting the findings of this study can be found within the article.

## Supplementary Materials

The following are available online at https://www.mdpi.com/article/10.3390/pathogens10121553/s1, Table S1. BLV proviral DNA prevalence in female beef cattle and dairy cows.
