# Supplementary material for: Potential Risk Factors Associated with Infection with Bovine Leukaemia Virus in Dairy and Beef Cattle in Taiwan
Source: Pathogens. 2021 Nov 29;10(12):1553. doi: 10.3390/pathogens10121553 (PMC8707763; doi:10.3390/pathogens10121553)
Supplement: Supplementary file 1 [file pathogens-10-01553-s001.zip › pathogens-1410732-supplementary.pdf]

Table S1. BLV proviral DNA prevalence in female beef cattle and dairy cows.

Factors	BLV detection					
	**Positive (n = 96)**	**Negative (n = 127)**	***p*-value**	**Odds Ratio**	**95% C.I.**	***p*-value**
**Function**						
Female beef	6	61	<0.001 §	Reference	5.655–33.987	<0.001¶
Lactating dairy	90	66		13.864		

Differences of analytes within groups and BLV positivity was performed with the §Chi-square test and ¶Logistic regression. BLV, bovine leukaemia virus. C.I., confidence interval. $p < 0.05$ indicates significant difference.
